# A survey of bluetongue infection in one-humped camels (*Camelus Dromedarius*); seroprevalence and risk factors analysis

**DOI:** 10.1186/s12917-022-03421-2

**Published:** 2022-08-22

**Authors:** Abdelfattah Selim, Roua A. Alsubki, Fatima M. Albohairy, Kotb A. Attia, Itoh Kimiko

**Affiliations:** 1 Department of Animal Medicine (Infectious Diseases), Faculty of Veterinary Medicine, Benha University, Toukh, 13736 Egypt; 2 Department of Clinical Laboratory Science, College of Applied Medical Sciences, King Saud University, , P.O. Box 2455, Riyadh, 11451 Saudi Arabia; 3 Department of Biochemistry, College of Science, King Saud University, P.O. Box 2455, Riyadh, 11451 Saudi Arabia; 4 Institute of Science and Technology, Niigata University, Ikarashi-2, Nishi-ku, Niigata, 950-2181 Japan

**Keywords:** Bluetongue, Sero-prevalence, Risk factors, Camels, Egypt

## Abstract

Bluetongue (BT) is an insect-borne, non-contagious viral disease which affects domestic ruminants including camels and is transmitted by *Culicoides* spp. Clinical symptoms of BT are typically seen in sheep, although subclinical BT infections are mostly seen in cattle, goats, and camelids. The goal of the present study was to evaluate the sero-prevalence of Bluetongue virus (BTV) in camels from some governorates in Egypt’s southern and northern regions, as well as the infection’s potential risk factors. During 2020–2021, a cross sectional study was conducted to screen presence of anti-BTV antibodies in 400 serum samples, which were collected randomly from camels, examined using competitive enzyme-linked immunosorbent assay (cELISA). The sera of 102 out of 400 camels tested positive for BTV, representing a frequency of 25.5%. Moreover, the odds of sero-positivity were higher among camels living in Aswan (OR = 5.33, 95%CI: 2.35–12.11), especially in females (OR = 2.63, 95%CI = 1.44–4.09) during summer season (OR = 2.40, 95%CI = 1.20–4.81). Furthermore, the probability of getting BTV infection increased when camels were exposed to the insect vectors (OR = 1.63, 95%CI = 0.87–3.09). The high prevalence of BTV in camels in several Egyptian regions highlights the need for more epidemiological investigations of BTV infection in other ruminant species in order to better control BT disease in these regions.

## Introduction

Bluetongue virus (BTV) is a double-stranded RNA virus that causes bluetongue disease (family *Reoviridae*, genus *Orbivirus*) with at least 29 identified serotypes that causes bluetongue in ruminants. The virus is transmitted from one susceptible host to another via different species of *Culicoides* biting midges. In Africa and southern Europe, *C. imicola* is the most common vector species [1]. Major susceptible hosts include sheep, goats, cattle, camels, llamas, deer, and antelopes. Clinical signs include fever, salivation, oral mucosa erosions, oedema of the cheeks and lips, erosions in/around nostrils, apathy, dysphagia, coronitis, lameness, conjunctivitis, muscular necrosis, and stiffness in the limbs. The clinical manifestation are severe in sheep, while cattle, goats, camelids, and wildlife typically show moderate symptoms [2].

The disease is classified as “notifiable” because of direct losses (death, reduction of milk production, sterility and/or abortion) and indirect losses (i.e. restrictions on foreign trade, survey and mass vaccination expenses, Control and treatment of vectors) [3–7].

According to Elzein [8], BTV infection is common among camels in Sudan, with a sero-prevalence of 16.6%, whereas Saeed and Aradaib [9] found that sero-prevalence of BTV in camels were extremely high (66.8%) in Khartoum State, Sudan. In Egypt, a few studies have been conducted to examine the overall state of BTV. Khaled et al. [10] recently found that BTV antibodies were common in sheep (23.2%) and goats (10.9%) in the Egyptian governorates of Aswan, Elwadi elgadid, Giza, and Marsa Matrouh. The main cause of BTV introduction into Egypt was thought to be the constant importation of viraemic ruminants or camels, mostly from Sudan [11]. BT is endemic in countries in Sub-Saharan Africa (including Sudan), with significant outbreaks occurring during periods of torrential rain and flooding [12]. Increased uncontrolled transboundary cattle movements could occur from socioeconomic changes, such as changes in meat market pricing, or changes in human population expansion and the resultant increased demand for meat [13]. As a result of all of these factors, the probability of BTV spreading into the Mediterranean basin and the Middle East has increased [14].

The prevalence of BTV infection in Egyptian camels is currently unknown, as well as the risk factors associated with it. Therefore, the goal of the present work was to conduct the first serological investigation of BTV infection and to investigate the associated risk factors in one-humped camels in some Egyptian governorates.

## Materials and methods

### Ethical statement

The study followed the Declaration of Benha University and was approved by the Ethics Committee of the Faculty of Veterinary Medicine (BUFVTM). All procedures were carried out in compliance with the ethical committee of the Faculty of Veterinary Medicine’s standards and regulations. The owners of the camels provided their verbal agreement for the samples to be taken. The study was conducted following ARRIVE guidelines.

### Study area

**Fig. 1:**
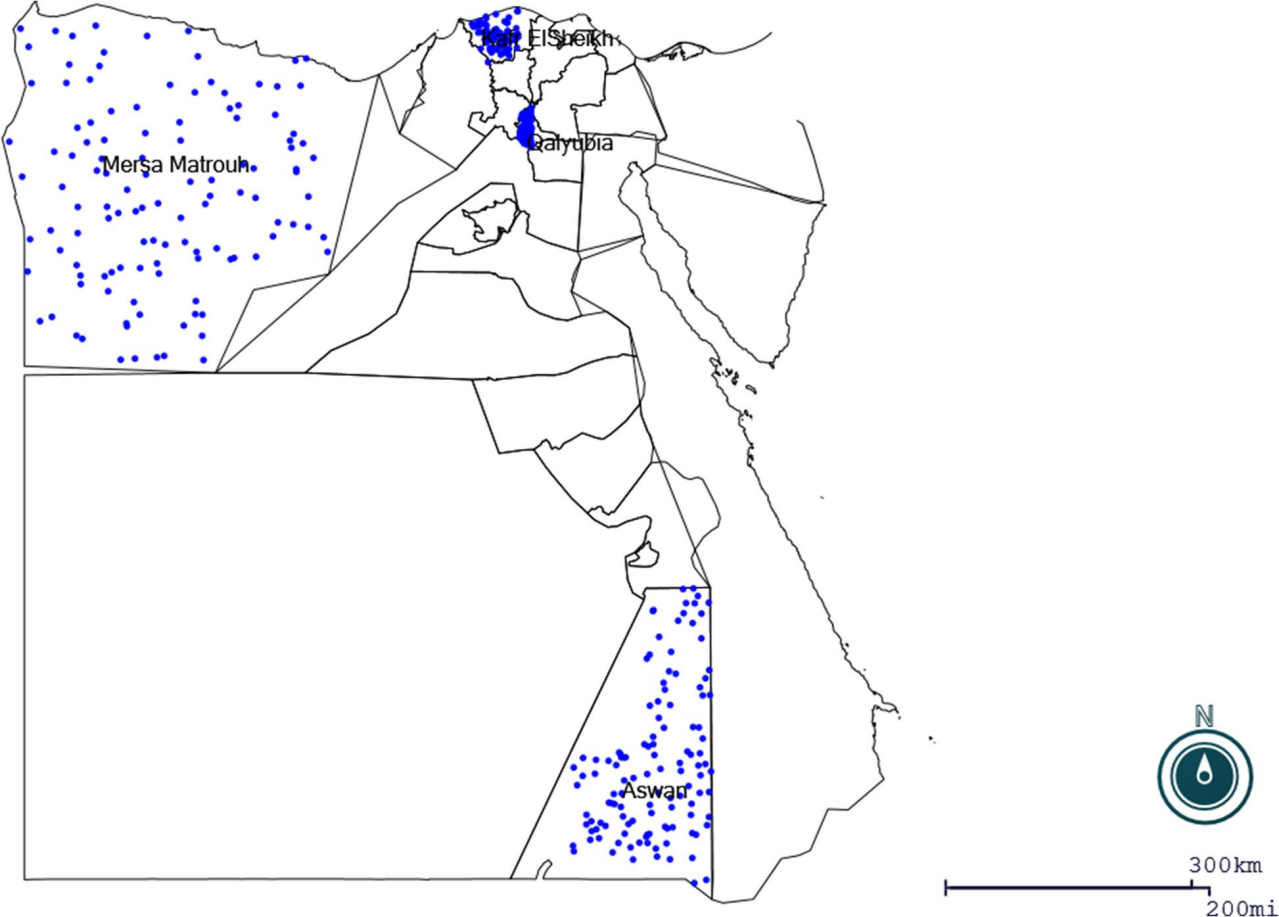
The study’s geographic locations and the number of camels investigated are represented by blue dots. MAP generated by EPI MAP (CDC)

This study was conducted in four governorates: Qalyubia, Kafr ElSheikh, Mersa Matrouh and Aswan which are located at 30°25 N to 31°13 E, 31.1107° N to 30.9388° E, 31°20′N 27°13′E and 24°05′20″N 32°53′59″E, respectively (Fig. 1).

The Köppen-Geiger climate classification for these governorates is BWh, which means “hot desert climate”. In the summer, the weather is very hot. The rainy season lasts 3 months, with yearly rainfall of 100 to 200 mm on average. The average annual temperature is between 20 °C and 45 °C throughout the year, with a relative humidity of approximately 43%. These governorates are one of the country’s major livestock growing zones, with large number of camels in particular. Therefore, the selected samples were representative. Moreover, Egypt shares borders with Sudan from south through Aswan governorate.

### Study design and blood sampling

This was a cross-sectional survey done between 2020 and 2021. A total of 400 samples were taken at random from 400 camels at the same time from all governorates under the study: two in the Nile Delta (Qalyubia and Kafr ElSheikh), one in the north portion (Mersa Matrouh), and one in the south (Aswan). The camels from each governorate were selected using simple random sampling. Data regarding to animal’s age (≤2, 2–5 and > 5 years), sex (male, female), season (spring, summer, winter, and autumn), and management risk factors such as herd size (≤50, ≥51), and insect vector (presence or absence) were all collected. Moreover, all of examined camels were non vaccinated against BTV. Blood was collected in plain vacuum tubes (without anticoagulant) and allowed to clot overnight at + 4 °C. After that, the serum was centrifuged for 15 minutes at 2000 rpm and stored at − 20 °C until serological examination.

### Serological analysis

A total of 400 serum samples were examined using IDEXX Bluetongue Competition Ab (IDEXX, USA). The procedures followed the manufacturer’s instructions. According to the manufacturer, the IDEXX cELISA kit has 100% specificity and 82.8% sensitivity. The optical density was read singly by AMR-100 reader (AllSheng, China) at 450 nm. When the optical density of the tested sera was less than or equal to 70% of the negative controls’ mean (S/N), they were classified as positive. Tested sera with an optical density more than or equal to 80% of the negative controls’ mean (S/N) were classified as negative, whereas those with an optical density less than 80% but greater than 70% of negative controls’ mean were classified as suspect and those animals were resampled and tested after 1 month.

### Statistical analysis

The statistical analysis was performed using SPSS version 24 (SPSS Inc., Chicago, U.S.A.). In a univariable analysis, chi-square test was performed to examine the risk factors’ relationships with BTV seroprevalence. Using multivariable logistic regression, potential risk factors having *p*-values < 0.2 were further evaluated. For the outcome variable, a multivariable model was performed using logistic regression analysis. Confidence interval (95%CI) and odds ratios (OR) were calculated. 

## Results

Out of 400 camels tested, 102 (25.5%) were shown to have antibodies to BTV and there were no clinical symptoms of BTV infection in any of camels. BTV sero-positivity had no statistically significant association with age or herd size (*P* > 0.05). The sero-prevalence rates were significantly different between different localities (*P* < 0.0001), whereas the highest prevalence rate for BTV was found in Aswan (42.5%), while the lowest prevalence rate was seen in Qalyubia (10.6%), Table 1.

**Table 1:** Univaraible analysis of Bluetongue infection in camels in relation to different variables

Variable	No of examined camels	No of test positives	No of negative	% of positive	95%CI	χ2statistic & *p*-value
**Locality**						
Qalyubia	85	9	76	10.6	5.6–18.9	32.844 d = 3 *P* < 0.0001*
Kafr ElSheikh	75	11	64	14.7	8.3–24.3	
Mersa matrouh	120	31	89	25.8	18.8–34.3	
Aswan	120	51	69	42.5	34–51.4	
**Age**						
≤ 2	50	14	36	28.0	17.4–41-6	2.539 d = 2 *P* = 0.28
> 2–5	190	54	136	28.4	22.4–35.2	
> 5	160	34	126	21.3	15.6–28.2	
**Sex**						
Male	180	29	151	16.1	11.4–22.2	15.186 d = 1 *P* < 0.0001*
Female	220	73	147	33.2	12.5–22.3	
**Herd size**						
≤ 50	240	57	183	23.8	18.8–29.5	0.967 d = 1 *P* = 0.32
≥ 51	160	45	115	28.1	21.7–35.5	
**Season**						
Autumn	84	25	59	29.8	21–40.3	24.322 d = 3 *P* < 0.0001*
Summer	138	52	86	37.7	30–46	
Winter	85	10	75	11.8	6.5–20.3	
Spring	93	15	78	16.1	10–24.9	
**Insect presence**						
Yes	290	85	205	29.3	24.3–34.8	8.059 d = 1 *P* = 0.005*
No	110	17	93	15.5	9.8–23.4	
Total	400	102	298	25.5	21.4–29.9	

*The result is significant at *P* < 0.05

The sero-prevalence was significantly (*P* < 0.0001) higher in females (33.2%) in comparison to males (16.1%), particularly among those infested with insects (29.3%). Moreover, BTV infection was significantly highest (*P* < 0.0001) during the summer season (37.7%), lowest during the winter season (11.8%), and average during the spring season (16.1%), Table 1. In addition, the median (> 2–5 years) and young ages groups (≤2 years) were more likely to be infected with BTV than older camels while the camels living in herd size (≥51) showed higher sero-positivity than those of small herds (≤50), Table 1.

In the present study, the camels living in Aswan were found more likely to be infected than those in Mersa Matrouh (OR = 5.33, 95%CI: 2.35–12.11, *P*-value = < 0.0001). Females were approximately three times more likely than males to be infected with BTV (OR = 2.63, 95%CI = 1.44–4.09, *P*-value = 0.001). Also, the presence of insects on camels increased the probability of BT sero-positivity by 1.63 times when compared to the absence of insects on animals (OR = 1.63, 95%CI = 0.87–3.09), Table 2.

**Table 2:** Multivariable logistic regression for risk factors associated with Bluetongue infection

Variable	OR	95% C.I. for OR		***P***-value
		Lower	Upper	
**Locality**				
Kafr ElSheikh	1.46	0.55	3.89	0.447
Mersa matrouh	2.31	0.99	5.33	0.051
Aswan	5.33	2.35	12.11	< 0.0001
**Sex**				
Female	2.63	1.44	4.09	0.001
**Season**				
Spring	1.65	0.75	3.61	0.211
Summer	2.40	1.20	4.81	0.013
Autumn	0.47	0.19	1.19	0.111
**Insect presence**				
Yes	1.63	0.87	3.06	0.130

*OR* Odds ratio, *CI* Confidence interval

Furthermore, the camels were more likely to contracting BTV infection particularly during summer season (OR = 2.40, 95%CI = 1.20–4.81, *P*-value = 0.013), Table 2.

## Discussion

Bluetongue produces significant financial losses and is a key source of concern for worldwide trade [15]. The severity of BTV infection ranges from completely subclinical to clinical, with the majority of infected camels showing no symptoms at all [16]. Furthermore, the global prevalence and form of BTV infection have altered dramatically in recent past. Individual BTV serotypes distribution is not consistent over the world, and it has changed dramatically as a result of climate change [17].

As a result of the high frequency of outbreaks among wild and domestic ruminants in formerly BTV-free areas, BTV has become a major veterinary issue for dairy producers, wildlife managers, and veterinary diagnosticians [18, 19]. However, in the Middle East and East Central Africa, including Egypt, relatively little information about BTV epidemiology is available. Moreover, absence of control measures for the disease (eg. vaccination, entomological or serological surveillance) in Egypt increase the possibility of disease spreading. We performed the present study to investigate the prevalence of BTV infection and its related risk factors among camels in certain governorates representing the north and south of Egypt, in order to progress beyond current knowledge of the disease’s epidemiology.

BTV sero-prevalence was found to be 25.5% overall in this work. Because Egypt does not have a BTV vaccination program, this sero-prevalence of BTV in some governorates could be attributed to spontaneous camel infection. BTV sero-positivity has been reported in dromedaries in numerous studies in different countries. The sero-prevalence rate of BTV among camels in this work lie in the same range that reported in previous study in Saudi Arabia (25.7%) by Yousef et al. [20]. Moreover, previous epidemiological studies found higher sero-prevalence for BTV in Sudan (78.6%) [21], Iran (93.5%) [22] and Turkey (88%) [23]. However, the overall prevalence of this study was greater than the previous reported rate in camels in the Kassala region in Sudan, with a prevalence of 4.3% [8].

In addition, the sero-prevalence was varied between different localities in the present study where the highest prevalence rate was found in South. This may be due to the co-existence of other species like as cattle, sheep, and goats with camel herds, as well as the favorable climatic circumstances that allow vectors to thrive in these areas [24–30].

In comparison, the Qalyubia governorate had the lowest infection rate (10%), this could be due to camels being separated from other animals, as well as the large camel population in this area [21]. It’s critical to stress the importance of the quantity and dispersion of *Culicoides* vector populations, as well as the composition of host species, climatic circumstances, and virus strains, may all influence sero-prevalence rates between countries [6, 31–34].

There was a significant relationship between BTV sero-positivity and sex when sex was considered a risk factor, with females having higher sero-prevalence than males. A similar findings were reported by Mahmoud et al. [35] in Libya.

Contrary to the present findings, Abraheem et al. [36] found equal sero-prevalence rate in both sexes with no significant disparity. The disparity in the present work could be related to disparities in male and female sample sizes, as well as variances in husbandry procedures [21, 36].

However, the risk assessment found no link between sero-prevalence and the age of the animal or the size of the herd. The findings are directly in line with previous findings reported in camels in Sudan [21, 36–38].

The results of the present study confirmed that the highest sero-prevalence of BTV was more common among herd ≥51when compared by ones ≤50, which are in accordance with findings reported by Saeed and Aradaib [9]. This could indicate that camels are more susceptible to infection when raised larger herds or in contact with more susceptible species such as cattle, sheep, and goats [39].

According to the findings, BTV sero-prevalence increased significantly during the summer season as compared to other seasons, especially in areas with a high insect population. This is consistent with previous findings of Elmahi et al. [21]. Such findings could be attributed to high density of *Culicoides* in these areas [18, 40], which plays an important role in transmission of BTV and its multiplication increased significantly during summer [38, 41, 42].

## Conclusion

The BTV antibodies have been detected in camels from Egypt. Moreover, the multivariable logistic regression analysis confirmed that the BTV seropositivity is strongly associated with locality, female sex, during summer season and in presence of insects. It is suggested that biting *Culicoides* midges engaged in BTV transmission be monitored using entomological methods. Additionally, studies on the ecology and epidemiology of BT in Egypt should be conducted in order to better predict the disease.

## Data Availability

All data generated or analysed during this study are included in this published article.
